# Are the effects of a non-drug multimodal activation therapy of dementia sustainable? Follow-up study 10 months after completion of a randomised controlled trial

**DOI:** 10.1186/1471-2377-12-151

**Published:** 2012-12-05

**Authors:** Katharina Luttenberger, Benjamin Hofner, Elmar Graessel

**Affiliations:** 1 Department of Medical Psychology and Medical Sociology, Friedrich-Alexander-Universität Erlangen-Nürnberg, Clinic for Psychiatry and Psychotherapy, Schwabachanlage 6, Erlangen 91054, Germany; 2 Department of Medical Informatics, Biometry and Epidemiology, Friedrich-Alexander-Universität Erlangen-Nürnberg, Waldstraße 6, Erlangen 91054, Germany

**Keywords:** Dementia, Non-drug-therapy, RCT, Follow-up study, Nursing home

## Abstract

**Background:**

Little is known about the long-term success of non-drug therapies for treating dementia, especially whether the effects are sustained after therapy ends. Here, we examined the effects of a one-year multimodal therapy 10 months after patients completed the therapy.

**Methods:**

This randomised, controlled, single-blind, longitudinal trial involved 61 patients (catamnesis: n = 52) with primary degenerative dementia in five nursing homes in Bavaria, Germany. The highly standardised intervention, MAKS, consisted of motor stimulation, practice of activities of daily living (ADLs), and cognitive stimulation. Each group of 10 patients was treated for 2 h, 6 days a week for 12 months. Control patients received standard nursing home care. At baseline, at the end of therapy (month 12), and 10 months thereafter (month 22), cognitive functioning was assessed using the cognitive subscale of the Alzheimer’s Disease Assessment Scale, and the ability to perform ADLs was assessed using the Erlangen Test of Activities of Daily Living.

**Results:**

During the therapy phase, the MAKS patients maintained their cognitive function and ability to carry out ADLs. After the end of therapy, both the control and the MAKS groups deteriorated in both their cognitive function (control, p = 0.02; MAKS, p < 0.001) and their ability to carry out ADLs (control, p < 0.001; MAKS, p = 0.001). However, in a confound-adjusted multiple regression model, the ability of the MAKS group to perform ADLs remained significantly higher than that of the control group even 10 months after the end of therapy (H_0_: β_MAKS_ + β_MAKS month 22_ = 0; χ^2^ = 3.8568, p = 0.0496). Cohen’s d for the difference between the two groups in ADLs and cognitive abilities 10 months after the end of therapy was 0.40 and 0.22, respectively.

**Conclusions:**

A multimodal non-drug therapy of dementia resulted in stabilisation of the ability to perform ADLs, even beyond the end of therapy. To prevent functional decline for as long as possible, therapy should be performed continuously until the benefit for the patient ends. Follow-up studies on larger numbers of patients are needed to definitively confirm these results.

**Trial registration:**

http://www.isrctn.com Identifier: ISRCTN87391496

## Background

Alzheimer’s dementia has been treated with drug therapy, non-drug therapy, and a combination of the two therapy types. In recent years, some studies have demonstrated the efficacy of non-drug therapy [1,2]. Unimodal interventions usually target cognition or the neuropsychological symptoms of dementia, and most available data are used to assess the efficacy of cognitive approaches [3]. However, the greatest effects of non-drug therapies in the treatment of Alzheimer’s dementia are achieved with multimodal therapy [1,4,5]. Cognitive training combined with treatment with acetylcholinesterase inhibitors has also been used and often brings better results than drug therapy alone [6,7], but intensive non-drug therapies alone also appear to be superior to drug therapy alone [7].

Little is known about the sustainability of any of these therapeutic effects. Whereas it would be expected that the cognitive performance of Alzheimer patients who respond to drug therapy will inevitably deteriorate when the medication is withdrawn [8,9], preliminary evidence indicates that non-drug therapies may result in greater sustainability of cognition and the ability to carry out activities of daily living (ADLs) [10,11]. Theoretically, this can be explained in that non-drug therapies strengthen abilities that the dementia patient can apply in everyday life and thus lead to a self-strengthening or more-or-less continued self-training. Yet no high-quality methodological studies have addressed this assumption. In a recent Cochrane review up to the end of 2011 [12] on the efficacy of cognitive training, 15 randomised controlled trials (RCTs) met the criteria for inclusion in the review. Only 4 of these studies examined the efficacy of the intervention after the end of therapy—3 during the first 3 months, and 1 at 10 months after the end of therapy. In the last study [6], neither cognition nor the ability to perform ADLs differed significantly between the therapy and control groups 10 months after the end of therapy [cognitive subscale of the Alzheimer’s Disease Assessment Scale (ADAS-cog): p = 0.66, SMD 0.12 (−0.41; 0.66); Texas Functional Living Scale: p = 0.12, SMD 0.43 (−0.30; 0.97)]. In addition to the 8-week cognitive training of the therapy group, all patients received the acetylcholinesterase inhibitor donepezil for the entire study period. In the re-analysis of the 3 other studies that examined the efficacy 1 to 3 months after the end of therapy, a significant effect on cognition was found (p = 0.05) [12]. These 3 studies did not examine ADLs. Further high-quality methodological studies of the sustainability of non-drug therapies are needed, not only because of their importance for cost savings in the health system [13].

In a blinded RCT design, we have demonstrated the efficacy of our multimodal activation therapy developed for institutionalised patients [14] with respect to cognition and the ability to carry out ADLs after a 12-month therapy [5,15]. In the subsequent follow-up study reported here in which we address the sustainability of the therapeutic effects, all of the patients still living 10 months after the end of therapy were re-examined using the same instruments.

## Methods

### Study design

Ten months after the end of our randomised, controlled, single-blind longitudinal trial of a multicomponent, non-pharmacological group therapy known as MAKS, we examined the efficacy of the therapy with dementia patients in five German nursing homes [5]. The MAKS therapy phase lasted 12 months, beginning in December 2008 and ending in December 2009. During this period, the MAKS group received 2h multimodal therapy 6 days a week, whereas a control group received the standard nursing home care. Both groups were examined with respect to cognitive function and their ability to perform ADLs prior to starting therapy (baseline), at the time therapy ended (month 12), and 10 months after the end of therapy (month 22).

### Sample

**Figure 1:**
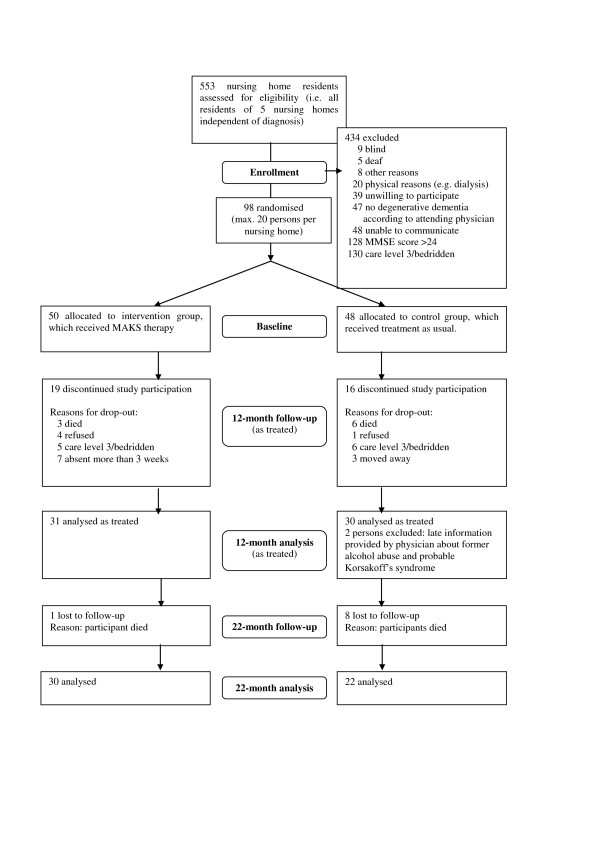
CONSORT flowchart.

Ninety-eight patients included in the original study fulfilled the following inclusion criteria: presence of primary degenerative dementia according to ICD-10 (F00, F03, or G30) and as confirmed by the attending physician; fewer than 24 points on the Mini-Mental State Examination (MMSE) [16]; and written informed consent of the patient or, when necessary, the patient’s legal guardian prior to baseline. This means that most of the patients are supposed to suffer from Alzheimer’s disease or a mixed form of Alzheimer/vascular dementia. Patients with pure vascular dementias were excluded. Of the approximately 400 residents who were positively screened for dementia, only 47 had to be excluded because of non-degenerative dementia according to the attending physician (see Figure 1). The sample therefore represents about 90% of the nursing home residents with cognitive deficits. The form sheet, all legal conditions, and the study design were examined by the Ethics Committee of the Medical Faculty of the University of Erlangen. Approval was granted on 10 July 2008 (Registration Number 3232). Exclusion criteria were as follows: vascular (F01) or secondary (F02) dementia according to ICD-10; the presence of other neurological/psychiatric disease(s) that could explain the patient’s decline in cognitive function; very high nursing care needs (i.e. care level 3, which is the highest level of the three-level scale currently used to determine eligibility for nursing care benefits in Germany); deafness; or blindness. Taking medication of any type did not affect inclusion or exclusion in our study (see Table 1 for medication taken). Of the 98 patients included, 35 met the dropout criteria during the intervention period, and 2 had to be excluded because of an incorrect diagnosis (i.e. n = 61 at month 12). Nine patients died in the 10 months between the end of therapy and the follow-up study. Hence, the follow-up analysis comprised 52 patients—22 in the control group and 30 in the MAKS intervention group (Figure 1).

**Table 1:** Characteristics of the patients participating through the follow-up analysis

**Characteristics**	**MAKS group**	**Control group**	**Total**
	**(n = 30)**	**(n = 22)**	**(n = 52)**
Age, mean (SD)	84.1 (5.02)	84.64 (5.45)	84.33 (5.16)
Women, no. (%)	27 (90.0)	16 (72.7)	43 (82.7)
Educational attainment, no. (%)			
No school completed	4 (13.3)	2 (9.1)	6 (11.5)
Lower secondary school (Hauptschule; through grade 9)	21 (70.0)	16 (72.7)	37 (71.2)
Secondary modern school (Realschule; through grade 10)	4 (13.3)	1 (4.5)	5 (9.6)
University-preparatory secondary school (Gymnasium; through grade 13)	0 (0)	1 (4.5)	1 (1.9)
Information lacking	1 (3.3)	2 (9.1)	3 (5.8)
Marital status, no. (%)			
Married	2 (6.7)	5 (22.7)	7 (13.5)
Widowed	22 (73.3)	16 (72.7)	38 (73.1)
Divorced	0 (0)	1 (4.5)	1 (1,9)
Single	6 (20.0)	0 (0)	6 (11.5)
MMSE mean (SD)	15.57 (4.83)	14.14 (5.45)	14.96 (5.1)
NOSGER subscale mood, mean (SD)	10.43 (3.13)	9.41 (2.99)	10.0 (3.08)
Charlson comorbidity index^a^, mean (SD)	0.95 (1.34)	1.0 (1.34)	0.97 (1.33)
Use of anti-dementia medications, no. (%)	3 (10.0)	3 (13.6)	6 (11.5)
Medication score^b^, mean (SD)	−1.57 (1.81)	−1.82 (1.87)	−1.67 (1.82)

Abbreviations: *MMSE*, Mini-Mental State Examination; *NOSGER*, Nurses’ Observation Scale for Geriatric Patients; *ADAS-cog*, Alzheimer’s Disease Assessment Scale, subscale cognition; *E-ADL test*, Erlangen Test of Activities of Daily Living.

^a^ Charlson comorbidity index: Effect of comorbidities (i.e. in addition to dementia) on mortality rate. A condition is assigned a score according to the mortality risk associated with it. One-year mortality increases from 12% (index = 0) to 85% (index ≥ 5) as the score increases.

^b^ Medication score: mean value of the sedating or activating (side-)effect of all medications taken by the patient, calculated using all prescribed medications, including anti-dementia drugs. To calculate these effects, two experts from the Department of Clinical Pharmacology at the University of Erlangen rated all medications in terms of their sedating or stimulating effect or side effects using a five-step scale: –2 (very sedating), –1 (sedating), 0 (neither sedating nor stimulating), +1 (stimulating), +2 (very stimulating).

### Patients

The number of patients analysed consisted of the number eligible at each time point (Figure 1). All patients who completed 12 months of either the MAKS therapy or the control group (standard nursing home care) were examined at the end of the therapy at month 12 (n = 61), and those who were still alive 10 months after the end of therapy were examined in the follow-up analysis at month 22 (n = 52). The patients who completed the study did not differ at baseline from those that were excluded in terms of age (t-test: p = 0.58), cognition (MMSE: p = 0.27; ADAS-cog: p = 0.14), and ability to carry out ADLs (Erlangen Test of Activities of Daily Living; E-ADL test: p = 0.08).

The characteristics of the 52 patients who completed the follow-up analysis are summarised in Table 1. Patients were on average 84 years old; 83% were female. Only 6 of the 52 patients were taking anti-dementia drugs; 3 were in the control group, and 3 were in the MAKS group. The MMSE score was on average 15 points.

### Treatment conditions

MAKS is a multicomponent group therapy consisting of tasks organised into three categories—motor stimulation (M), ADLs (A), and cognition (K)—preceded by a short introduction consisting of what we called a spiritual element (S). Each daily session began with this introduction, which lasted approximately 10 min and was designed to help the dementia patients feel part of the group. This was followed by about 30 min of motor exercises. After a 10-min break, the patients spent approximately 30 min completing a variety of cognitive tasks. This was followed by about 40 min of ADLs [for further information, see [5]].

The members of the control group received the standard care offered in each nursing home and were free to participate in any of the regular, non-MAKS activities offered at the nursing home. Patients in the control group participated in an average of two of these non-MAKS activities per week. Also, patients in the MAKS group were free to take part in these non-MAKS activities in addition to MAKS and did so once a week on average. The study did not interfere in any way with the patients’ existing pharmacological treatment or nursing care.

### Implementation of treatment

MAKS therapy was conducted in each nursing home by two therapists and one aide from Monday to Saturday from 9:30 am to 11:30 am for 12 months. The therapists were registered geriatric nurses. Each therapy group consisted of 10 dementia patients. Therapists and aides received a standardised handbook from the central study site describing in detail the steps to be taken on each day of therapy [14]. This guaranteed that the same tasks would be performed on any given day at each nursing home [for further information, see [5]].

### Instruments and data recording

The cognitive and ADL-abilities of the patients were recorded by independent evaluators, who were blinded to treatment allocation and were not part of the nursing home staff. The patients were evaluated before the therapy commenced (baseline), at the end of the 12-month therapy (month 12), and 10 months after completion of the therapy (month 22), with all patients having the standard nursing home care during this latter period. Data were pseudonymised and submitted to the central study site.

Cognitive abilities were measured using the ADAS-cog [17]. The scale ranges from 0 to 70, with higher scores indicating greater deficits (Cronbach’s α = 0.82; construct validity, correlation with MMSE −0.81).

The ability to carry out ADLs was measured using the E-ADL test [18]. This is a performance test of fundamental abilities of daily living under standardised conditions and includes pouring a drink, cutting a piece of bread, opening a little cupboard, washing one’s hands, and tying a bow. The range is from 0 to 30, with higher scores indicating a greater ability to carry out the activities (α = 0.77).

Study coordinators recorded each patient’s age, gender, educational attainment, family status, and nursing care needs at baseline. The nursing staff rated symptoms of depression among the patients at baseline using the mood subscale of the Nurses’ Observation Scale of Geriatric Patients (NOSGER) [19] (test-retest reliability: 0.85; correlation with the Geriatric Depression Scale: r_S_ = 0.63).

We also calculated the effect of any previous medical diagnoses on the mortality rate using the Charlson comorbidity index [20]. Potential bias resulting from pharmacological interventions was accounted for by using a medication score (sedative/stimulating effect of all medication) (see Table 1).

### Statistical analysis

We statistically analysed the data of all patients who completed the primary intervention period of 12 months either in the MAKS group or in the control group (n = 61). In the analysis of the follow-up data, we made attempts to record outcome variables for all patients who completed the intervention period and who were still alive 10 months after the end of therapy (n = 52). If more than 20% of the items on the ADAS-cog subscale or the E-ADL test were missing for any given patient (e.g. because of his or her refusal to complete the test), the score was calculated according to the expectation-maximum (EM) algorithm. Under these conditions, imputation at the 10-month follow-up was necessary in 4 cases. The scores for patients who died during the 10-month interim period without therapy (n = 9) were not imputed. The number of patients eligible for the analysis in the multiple regression models was 61 at the end of the therapy (at month 12) and 52 after the 10 months following the therapy (at month 22).

To describe the course, t-tests for dependent samples were calculated for both groups for the two time periods. The first period was the 12-month therapy period (from baseline to month 12), and the second period was the 10-month interim without therapy between evaluations (from month 12 to month 22). Model diagnostics of the differences revealed no deviation from a normal distribution. Cohen’s d with pooled standard deviation [21] was calculated as the measure of effect size.

Differences between the groups were calculated with multiple analyses to adjust for possible confounders; multiple linear mixed models were computed. We computed two separate models, one for the E-ADL test score and one for the ADAS-cog score. For the analysis, we adjusted the score at months 12 and 22 for the baseline values (score at month 12 – baseline score, and score at month 22 – baseline score). We then built a variable that contains both measurements for each patient (where available) and used another variable to reflect the time of measurement (“repeated measurements”). We included the following independent variables: intervention group (MAKS vs. control), observation time (month 12 vs. month 22), age of the patient, medication score, NOSGER subscale mood, anti-dementia medication, and the interaction of the intervention group and observation time to account for possible changes of the group effect over time. The “nursing home” entered the model as a random effect. Model diagnostics revealed no substantial deviation from the model assumptions.

In a sensitivity analysis, we additionally included a random slope for time to account for possible non-systematic variations over time. For the analyses, we used the statistical software packages R [22] and SPSS [23]. P-values smaller than 0.05 indicate significant effects.

## Results

**Figure 2:**
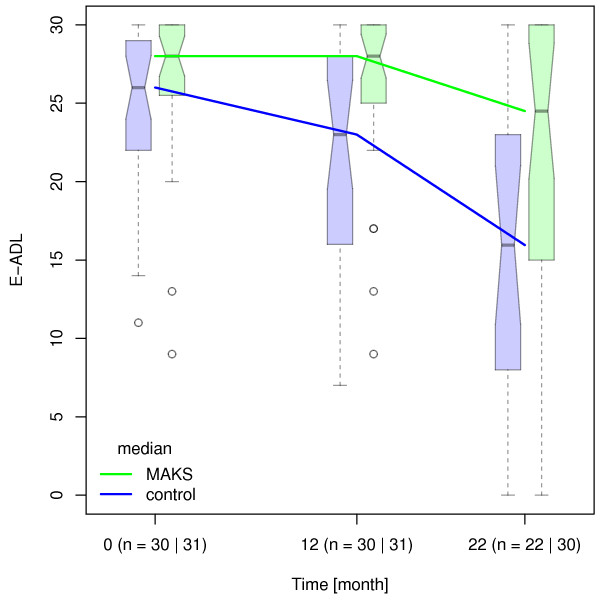
**Median E-ADL test values over time.** Median E-ADL test values in the groups MAKS and control over time together with the corresponding notched boxplots. Lower scores indicate greater deficits. Boxplots represent the distribution of raw data values. Non-overlapping notches are a (rough) indicator of significantly different medians [see [28].

**Figure 3:**
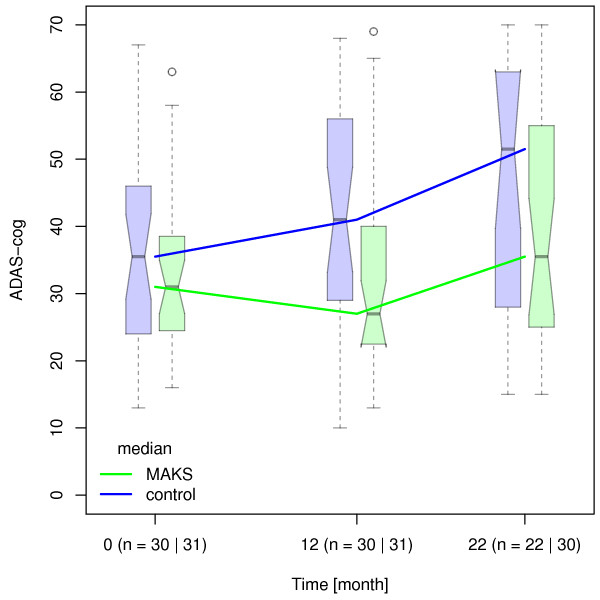
**Median ADAS-cog values over time.** Median ADAS-cog values in the MAKS and control groups over time together with the corresponding notched boxplots. Higher scores indicate greater deficits. Boxplots represent the distribution of raw data values. Non-overlapping notches are a (rough) indicator of significantly different medians [28].

In the course of the 12 months of therapy, the cognitive and ADL-abilities of the patients in the control group (n = 30) decreased significantly (t-test for dependent samples; ADAS-cog: baseline, 35.6 ± 14.8 SD; at month 12, 40.8 ± 17.0, p = 0.039; E-ADL test: baseline, 24.3 ± 5.6; at month 12, 21.5 ± 7.4, p = 0.005). By contrast, these abilities of the MAKS group (n = 31) remained stable during this time (ADAS-cog: baseline, 32.6 ± 11.5; at month 12, 32.5 ± 15.3, p = 0.99; E-ADL test: baseline, 26.6 ± 5.1; at month 12, 26.3 ± 14.8, p = 0.72; see also [5]). The cognitive and ADL-abilities of patients in both groups (n = 52) deteriorated significantly between the end of therapy and the follow-up evaluation after 10 months (MAKS group: ADAS-cog: 40.06 ± 17.04, p = <0.001; E-ADL test: 20.90 ± 10.08, p = 0.001 / control group: ADAS-cog: 46.92 ± 18.73, p = 0.015; E-ADL test: 15.38 ± 9.43, p < 0.001) (Figures 2 and 3). At the time of the follow-up evaluation 10 months after the end of therapy, Cohen’s d was 0.40 for ADL-abilities and 0.22 for cognitive abilities.

**Figure 4:**
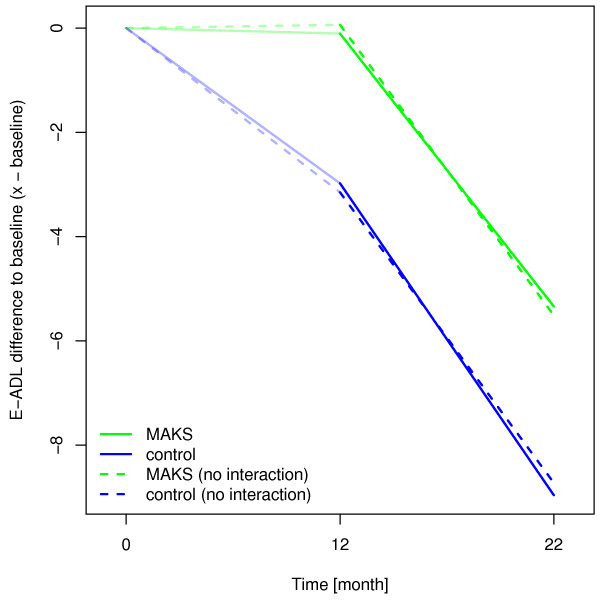
**Estimated changes of E-ADL test score compared to baseline using a random effects model.** Higher scores indicate better abilities to perform ADLs. The solid lines represent effect estimates from a model with an interaction term for time and group, i.e. where the group effect can change over time. The dashed lines indicate a model without interaction. In this case, the group effect is forced to stay constant (as can be seen from the parallel dashed lines from month 12 to month 22).

In the confound-adjusted multiple regression model with the random effect of “nursing home” (see Additional file 1), the E-ADL test score of the MAKS group remained significantly higher than that of the control group even 10 months after the end of therapy (H_0_: β_MAKS_ + β_MAKS month 22_ = 0; χ^2^ = 3.86, p = 0.0496). The effect of therapy on the ability to carry out ADLs over time was not only sustainable but even increased slightly over time after the end of the MAKS therapy, as can be seen in the widening of the solid lines in Figure 4.

In a sensitivity analysis, we additionally adjusted for a potential auto-correlation between the time points and thus included a random slope for time. In this case, the effect of MAKS therapy 10 months after the end of therapy was no longer significant (χ^2^ = 2.49, p = 0.116). Despite the lack of a significant difference in this case, the effect estimates revealed the same structure as depicted in Figure 4, i.e. the effects of the model with the interaction indicate that the effect of therapy (month 12) was not only preserved but had a slight tendency to become even more pronounced 10 months after the therapy (month 22).

**Figure 5:**
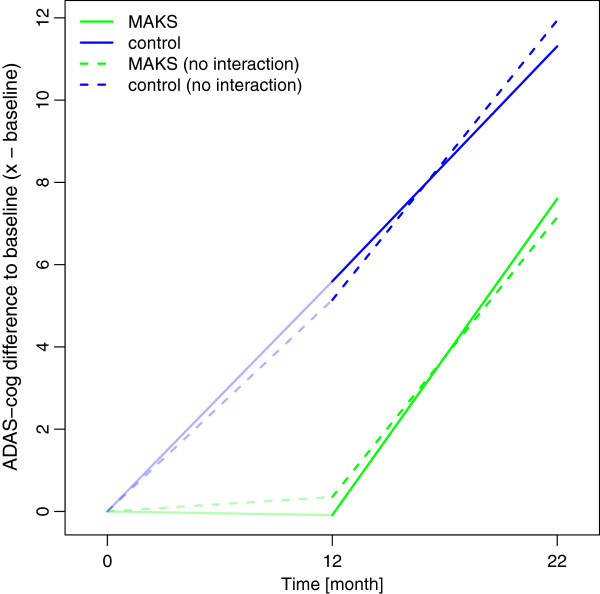
**Estimated changes of ADAS-cog compared to baseline using a random effects model.** Higher scores indicate greater deficits. The solid lines represent effect estimates from a model with an interaction term for time and group, i.e. where the group effect can change over time. The dashed lines indicate a model without interaction. In this case, the group effect is forced to stay constant (as can be seen from the parallel, dashed lines from month 12 to month 22).

As for the ADLs, we used a confound-adjusted model with a random effect of “nursing home” to evaluate the long-term effect of MAKS therapy on cognition (ADAS-cog; see Additional file 2). We did not observe a lasting effect of MAKS therapy on ADAS-cog (H_0_: β_MAKS_ + β_MAKS month 22_ = 0; χ^2^ = 1.15, p = 0.282). One can even deduce from Figure 5 that the groups were slowly converging.

The sensitivity analysis with an additional random slope of time did not change the result (MAKS vs. control at month 22: χ^2^ = 0.88, p = 0.347).

## Discussion

The present study addressed whether the positive effects on cognition and the ability to carry out ADLs of a 12-month multimodal therapy of dementia patients are sustainable 10 months after the end of therapy. This study is thus one of the first RCTs to systematically examine the sustainability of the effects of a non-drug therapy in dementia patients. Our results using a confound-adjusted multiple regression model indicated that the positive effects of the group therapy (i.e. therapy group vs. control group) on the ability to carry out ADLs were indeed sustainable. This effect was still existent but no longer statistically significant when controlled for a random subject-specific slope. Such a finding is unique in the literature. To date, only two randomised studies have addressed the sustainability of non-drug therapy procedures on cognition and the ability to carry out ADLs, but in combination with cholinesterase inhibitors.

In a recent study on dementia patients living at home, Giordano et al. [24] showed that the success of therapy combining cholinesterase inhibitors and a 3-week Reality Orientation Training (ROT) could still be demonstrated 2 months after the active intervention phase as long as family members continued the ROT. Control patients were treated only with cholinesterase inhibitors. The patients of the therapy group profited, however, only in the cognitive area; effects on their ability to carry out activities of daily living were never observed. Moreover, therapy was never withdrawn as medication was maintained in both the ROT and control groups, and caregivers of the test group were asked to continue ROT at home, even if this potentially occurred in a less systematic form. One problem that this study had is the short time frame, as even placebo effects can last up to 9 months as Ito et al. [25] demonstrated in a meta-analysis.

Chapman et al. [6] studied the efficacy of a combination therapy consisting of donepezil and cognitive stimulation therapy in a controlled randomised study of 54 dementia patients. The control group received donepezil alone. The parameters were cognition, ability to carry out ADLs, and neuropsychiatric symptoms. The therapy was conducted once a week for 2 months. With the two scales used (ADAS-cog and Texas Functional Living Scale TFLS), which are comparable to those of our study, the authors found a significant decline in abilities in both the test and control groups over the course of a year. No effects on cognition or the ability to perform ADLs could be demonstrated at the end of therapy or 10 months after ending therapy. However, an advantage of the combined therapy was found in the MMSE and some subscales of the Neuropsychiatric Inventory (NPI) 10 months after the end of therapy. As effects of pharmacological treatment have been shown elsewhere to last less than 12 months [25], the differences between the treatment and control groups are more likely related to the cognitive stimulation. The lack of effects on the ADAS-cog and the TFLS of this therapy may be attributable to its low intensity (8 sessions over 2 months), which is considerably less intensive than our MAKS therapy (about 300 sessions over 12 months). Other studies examining the sustainability of non-drug therapy procedures have usually addressed the neuropsychological symptoms of dementia, such as mood and behaviour e.g. [26,27] or are of limited reliability due to the small number of patients [10,11].

In the main analysis, we found a long-term effect of the MAKS therapy on the ability to carry out ADLs but not on cognition. Future studies should test whether ADL training will preserve the self-reliance of the participating home residents more than that of the control group. This in turn would mean that ADLs are performed independently to a greater extent and are thus automatically trained further. By contrast, the long-term effect of MAKS therapy on cognitive functions is much lower. Here, the difference between the control group and therapy group was smaller 10 months after the end of therapy, and the effect of the therapy was no longer demonstrable in multivariate analyses. Compared to a strictly pharmaceutical therapy, a non-drug therapy thus appears to offer the potential of an effect that promotes independence in everyday living that lasts beyond the term of therapy, even if the positive effect on cognitive functions did not continue after therapy ended. Because the cognitive abilities as well as ADLs abilities were preserved in the MAKS group during therapy, a continuous therapy could perhaps stabilise both abilities even longer.

A limitation of the present study is the number of patients. After 22 months, only a relatively small sample number of patients remained (n = 52), owing in part to the high mortality rate in the age spectrum examined. The small number of cases is probably also the reason why the model in the sensitivity analysis with a random slope did not attain significance for the ability to carry out ADLs, especially since the variation of the measured E-ADL test scores increased over time (see Figure 2). Also, results are valid only for patients with degenerative dementias such as Alzheimer’s dementia or dementia of the mixed form. Effects on persons with vascular dementia were not examined. Another limitation might be the use of the ADAS-cog as the outcome instrument for cognition. The ADAS-cog does not have parallel versions for test-retest settings; stabilisation might therefore be due to learning effects rather than due to therapy. However, this effect would be the same in the control and therapy groups. Thus, differences between the groups cannot be explained by learning effects. Further studies with a considerably larger number of patients, an extended follow-up period, and more sensitive test items are thus necessary to verify the results shown here.

One strength of the study is the strict RCT design used during the therapy phase. To our knowledge, our study is thus the first to examine the sustainability of an exclusively non-drug therapy of dementia after completion of the active therapy phase. Our method is also notable in that all dependent variables were recorded blindly by testing and not, as is often the case, by using outside assessment.

## Conclusions

Our intensive multimodal therapy was elsewhere shown to be effective in the treatment of dementia with regard to both cognitive functioning and the ability to perform ADLs with greater effect sizes for patients with mild to moderate dementia [5]. This study of the long-term effects of a non-drug therapy showed that upon withdrawal of non-drug therapy, the ability to perform ADLs—as compared to cognitive functioning—tended to be more focused on “self-preservation”. Hence, intensive multimodal therapy should be started as early as possible to maintain the abilities still present in the dementia patient. It is also especially important to apply the therapy continuously to retain for as long as possible not only the ADL-abilities but also the cognitive functions, and thus to retain the independence of people with dementia. Future research on this topic including a larger sample size, even longer follow-up periods, and perhaps different outcome measurements should be performed to support the findings.

## Abbreviations

ADLs: Activities of daily living; RCT: Randomised controlled trial; ADAS-cog: Cognitive subscale of the Alzheimer’s Disease Assessment Scale; MMSE: Mini-Mental State Examination; E-ADL: Erlangen Test of Activities of Daily Living; NOSGER: Nurses’ Observation Scale of Geriatric Patients; EM: Expectation-maximimum; ROT: Reality Orientation Training; TFLS: Texas Functional Living Scale; NPI: Neuropsychiatric Inventory.

## Competing interests

The authors declare that they have no competing interests. The research reported was funded by the German Ministry of Health (LT-Demenz-44-059), which had no role in designing or conducting the study; in collecting, analysing, or interpreting the data; or in preparing, reviewing, or approving the manuscript. The researchers acted fully independently of the sponsor.

## Authors’ contributions

All authors had full access to all of the data in the study. EG designed the study concept and obtained funding. KL and BH provided administrative, technical, or material support. KL and EG acquired data. All authors statistically analysed and interpreted the data, critically revised the manuscript for important intellectual content, and read and approved the final manuscript.

## Pre-publication history

The pre-publication history for this paper can be accessed here:

http://www.biomedcentral.com/1471-2377/12/151/prepub

## Supplementary Material

Additional file 1 **Table S2.** Fixed effects of mixed-effects model with E-ADL test as dependent variable and “nursing home” as random effect. Click here for file

Additional file 2 **Table S3.** Fixed effects of mixed effects model with ADAS-cog as dependent variable and “nursing home” as random effect. Click here for file
