# Supplementary material for: Are the effects of a non-drug multimodal activation therapy of dementia sustainable? Follow-up study 10 months after completion of a randomised controlled trial
Source: BMC Neurol. 2012 Dec 5;12:151. doi: 10.1186/1471-2377-12-151 (PMC3527171; doi:10.1186/1471-2377-12-151)
Supplement: Additional file 1 — Table S2. Fixed effects of mixed-effects model with E-ADL test as dependent variable and “nursing home” as random effect. [file 1471-2377-12-151-S1.doc]

**Table 2 – Fixed effects of mixed-effects model with E-ADL test as dependent variable and “nursing home” as random effect.**

The difference between the MAKS and control groups 10 months after the end of therapy is given by the combination of the group effect and the interaction effect (βMAKS + βMAKS month 22).

| Independent variables | Unstandardised β (95% CI) | Std. Error | t value | p value |
| --- | --- | --- | --- | --- |
| Group (control = 0 vs. MAKS = 1)* | 2.87 (−0.44, 6.19) | 1.69 | 1.70 | 0.093 |
| Time (month 12 = 0 vs. month 22 = 1) | −5.98 (−9.53, −2.44) | 1.81 | −3.31 | 0.001 |
| Age | 0.01 (−0.23, 0.25) | 0.12 | 0.07 | 0.943 |
| Gender (female = 0 vs. male = 1) | −0.09 (−3.40, 3.22) | 1.69 | −0.05 | 0.957 |
| Medication score | 0.04 (−0.67, 0.76) | 0.36 | 0.12 | 0.908 |
| NOSGER, mood | −0.45 (−0.86, −0.03) | 0.21 | −2.12 | 0.037 |
| Use of anti-dementia medication | −2.07 (−6.18, 2.05) | 2.10 | −0.98 | 0.327 |
| Interaction group and time (MAKS at month 22 = 1)^1^ | 0.75 (−4.04, 5.53) | 2.44 | 0.31 | 0.760 |
| (Intercept) | 1.00 (−20.40, 22.39) | 10.92 | 0.09 | 0.927 |

^*^MAKS therapy had a positive effect, i.e. it increased the E-ADL test score (better ADL abilities); at month 22, this effect was further increased by the positive effect of the interaction.
