# Supplementary material for: Are the effects of a non-drug multimodal activation therapy of dementia sustainable? Follow-up study 10 months after completion of a randomised controlled trial
Source: BMC Neurol. 2012 Dec 5;12:151. doi: 10.1186/1471-2377-12-151 (PMC3527171; doi:10.1186/1471-2377-12-151)
Supplement: Additional file 2 — Table S3. Fixed effects of mixed effects model with ADAS-cog as dependent variable and “nursing home” as random effect. [file 1471-2377-12-151-S2.doc]

**Table 3 – Fixed effects of mixed effects model with ADAS-cog as dependent variable and “nursing home” as random effect.**

The difference between the MAKS and control groups 10 months after the end of therapy is given by the combination of the group effect and the interaction effect (βMAKS + βMAKS month 22).

|  | Unstandardised β (95% CI) | Std. Error | t value | p value |
| --- | --- | --- | --- | --- |
| Group (control = 0 vs. MAKS = 1) | −5.68 (−11.90, 0.53) | 3.17 | −1.79 | 0.076 |
| Time (month 12 = 0 vs. month 22 = 1) | 5.72 (−0.91, 12.34) | 3.38 | 1.69 | 0.094 |
| Age | 0.32 (−0.13, 0.77) | 0.23 | 1.38 | 0.170 |
| Gender (female = 0 vs. male = 1) | −0.20 (−6.47, 6.08) | 3.20 | −0.06 | 0.951 |
| Medication score | −0.72 (−2.06, 0.62) | 0.68 | −1.06 | 0.294 |
| NOSGER, mood | 0.75 (−0.03, 1.52) | 0.40 | 1.89 | 0.062 |
| Use of anti-dementia medication | 2.29 (−5.42, 9.99) | 3.93 | 0.58 | 0.562 |
| Interaction group and time (MAKS at month 22 = 1) | 1.98 (−6.97, 10.92) | 4.56 | 0.43 | 0.665 |
| (Intercept) | −30.34 (−70.70, 10.02) | 20.59 | −1.47 | 0.144 |

* MAKS therapy had a negative effect, i.e. it reduced the ADAS-cog (less cognitive impairment); at month 22, this effect was decreased by the positive effect of the interaction.
